# Comparative Effects of Basic Fibroblast Growth Factor Delivery or Voluntary Exercise on Muscle Regeneration after Volumetric Muscle Loss

**DOI:** 10.3390/bioengineering9010037

**Published:** 2022-01-14

**Authors:** Caroline Hu, Bugra Ayan, Gladys Chiang, Alex H. P. Chan, Thomas A. Rando, Ngan F. Huang

**Affiliations:** 1 Veterans Affairs Palo Alto Health Care System, Palo Alto, CA 94304, USA; chu@pavir.org (C.H.); gchiang@pavir.org (G.C.); ahpchan@stanford.edu (A.H.P.C.); rando@stanford.edu (T.A.R.); 2 Department of Cardiothoracic Surgery, Stanford University, Stanford, CA 94305, USA; bugra@stanford.edu; 3 Stanford Cardiovascular Institute, Stanford University, Stanford, CA 94305, USA; 4 Department of Neurology, Stanford University, Stanford, CA 94305, USA; 5 Department of Chemical Engineering, Stanford University, Stanford, CA 94305, USA

**Keywords:** volumetric muscle loss, basic fibroblast growth factor, nanofibrillar scaffold, collagen, rehabilitative exercise, regeneration, mouse

## Abstract

Volumetric muscle loss (VML) is associated with irreversibly impaired muscle function due to traumatic injury. Experimental approaches to treat VML include the delivery of basic fibroblast growth factor (bFGF) or rehabilitative exercise. The objective of this study was to compare the effects of spatially nanopatterned collagen scaffold implants with either bFGF delivery or in conjunction with voluntary exercise. Aligned nanofibrillar collagen scaffold bundles were adsorbed with bFGF, and the bioactivity of bFGF-laden scaffolds was examined by skeletal myoblast or endothelial cell proliferation. The therapeutic efficacy of scaffold implants with either bFGF release or exercise was examined in a murine VML model. Our results show an initial burst release of bFGF from the scaffolds, followed by a slower release over 21 days. The released bFGF induced myoblast and endothelial cell proliferation in vitro. After 3 weeks of implantation in a mouse VML model, twitch force generation was significantly higher in mice treated with bFGF-laden scaffolds compared to bFGF-laden scaffolds with exercise. However, myofiber density was not significantly improved with bFGF scaffolds or voluntary exercise. In contrast, the scaffold implant with exercise induced more re-innervation than all other groups. These results highlight the differential effects of bFGF and exercise on muscle regeneration.

## 1. Introduction

Volumetric muscle loss (VML) is characterized by irreversible damage in skeletal muscle structure and function due to the loss of a significant portion of skeletal muscle. Traumatic injuries leading to VML are associated with impaired endogenous muscle regeneration, long-term disability, and cosmetic deformities [1,2]. Current surgical approaches to treat VML by muscle flap grafting or scar tissue debridement are associated with significant donor site morbidity and functional deficiency [1,3,4,5].

Skeletal muscle is generally composed of bundles of parallel-aligned myofibers, interspersed with blood vessels within close proximity [6] that provide nutrients and oxygen to the myofibers. Muscle regeneration is a highly dynamic process that involves the organized orchestration of growth factor elaboration. Among the growth factors involved in muscle regeneration, basic fibroblast growth factor (bFGF) is elaborated upon injury to stimulate the activation and proliferation of muscle satellite cells to orchestrate muscle regeneration [7,8]. Given the importance of bFGF to muscle regeneration, the provision of bFGF may be an important component of the treatment strategy for VML.

The promise of regenerative medicine is the full regeneration of damaged tissues, either by promoting repair from endogenous stem cells or by the transplantation of cells to enhance regeneration [9]. To date, only a limited number of biological approaches have been employed for the treatment of VML. Implantation of decellularized scaffolds into sites of VML has been shown to induce de novo formation of skeletal muscle, in part due to the structural support provided by the scaffold [1]. The addition of myogenic cells seeded in decellularized extracellular matrix (ECM) scaffolds was shown to improve force generation and muscle regeneration at the site of VML [10,11]. Although decellularized scaffolds are commercially available and have been shown to be safe in clinical studies [12], a major disadvantage of decellularized scaffolds is the limited tunability of their mechanical properties and limited efficacy to date. In contrast, scaffolds derived from reconstituted ECM proteins or synthetic polymers offer greater tunability of mechanical properties, including controllable porosity, scaffold structure, and a degree of crosslinking. Scaffolds derived from reconstituted collagen or fibrin have been previously tested for safety in the setting of VML [13,14,15]. To promote muscle formation that recapitulates the highly organized native muscle structure, we previously showed that spatially patterned scaffolds provide physical guidance cues to direct muscle organization and function [16]. Parallel-aligned nanopatterned collagen scaffolds not only modulated cellular organization but also biological processes that include cell migration, angiogenesis, proliferation, and cell survival [17,18,19,20,21]. In the setting of VML, spatially nanopatterned collagen scaffolds guided the organization of newly formed myofibers that, in parallel, better mimics the physiology of native muscle [16]. These findings concur with other published studies stating that spatially patterned biomaterials regulate cellular alignment, function, and tissue morphogenesis [21,22,23,24].

Recent studies suggest that rehabilitative exercise may be beneficial for the treatment of VML [13,25]. In particular, voluntary wheel running exercise was shown to improve force transmission in regenerated muscle after VML [25]. We have previously demonstrated that a voluntary wheel exercise regimen after the implantation of decellularized scaffolds seeded with muscle satellite cells resulted in the formation of more mature neuromuscular junctions, greater force production, and increased revascularization, when compared to implantations of bioconstructs in the absence of exercise [26]. Decellularized scaffold implantation into injured human muscle with exercise intervention showed improved functional outcomes in strength and range of motion [27]. More recently, we have demonstrated that the implantation of off-the-shelf spatially nanopatterned collagen scaffolds, in conjunction with voluntary exercise, could stimulate vascular perfusion and neuromuscular junction formation [13]. Furthermore, we showed that the combination of implanted nanopatterned scaffolds with the pro-regenerative release of insulin-like growth factors in conjunction with rehabilitative exercise could promote both revascularization and re-innervation [28]. Based on these studies, rehabilitative strategies appear to provide important mechanical signaling cues to enhance aspects of muscle regeneration after the implantation of biomaterials and soluble growth factors.

Since rehabilitative exercise and bFGF elaboration are two potential strategies for the treatment of VML, the objective of this study was to compare the effects of implanted spatially nanopatterned bFGF-releasing scaffolds to that of implanted scaffolds in conjunction with voluntary caged wheel exercise. By evaluating muscle physiology and tissue histology in a mouse model of VML, we show that bFGF and voluntary exercise have differential therapeutic benefits for the treatment of VML.

## 2. Materials and Methods

### 2.1. Fabrication of Aligned Nanofibrillar Collagen Scaffolds

Aligned nanofibrillar collagen scaffolds were fabricated as described in our previous work [13,16,28]. In brief, rat-tail collagen-type I (Corning, Corning, NY, USA) was dialyzed to 30 mg mL^−1^ in 0.02 N in acetic acid (pH 3.5) and then extruded from a 22 G blunt tip needle onto glass slides that were submerged in phosphate-buffered saline (10× PBS, pH 7.4) at 37 °C, leading fibrillogenesis along the direction of extrusion. To fabricate parallel-aligned 3D scaffold bundles, 16 scaffolds strips were intrinsically sticky and adhered in parallel to one another before immobilizing the scaffold bundle onto surface-reactive Nexterion H (Schott, Jena, Germany) slides. The scaffolds were washed with 1× PBS, dried overnight in a laminar flow hood, and then disinfected with 70% ethanol.

In order to prepare growth factor-laden scaffolds, the scaffolds were submerged within a solution of recombinant human bFGF (100 μg mL^−1^, Peprotech, Cranbury, NJ, USA) overnight at 37 °C and 5% CO_2_. Control scaffolds were incubated in 0.1% bovine serum albumin (BSA, Sigma, St. Louis, MO, USA). After overnight incubation, scaffold bundles were removed from the Nexterion H Slides and then trimmed to 4 mm × 3 mm × 2 mm scaffolds for implantation or in vitro studies. The surface topography of the scaffolds was assessed by routine scanning electron microscopy, based on previous methods [20,29,30].

### 2.2. Quantification of bFGF Release

To determine the kinetics of bFGF release from the scaffolds over time, the bFGF-laden scaffolds or control scaffolds were incubated in 0.1% BSA. At time points over 21 days, the supernatant was sampled and then replaced with fresh BSA. The release of bFGF was quantified by an enzyme-linked immunosorbent assay (ELISA) using a Human bFGF Quantikine ELISA Kit (R&D Systems, Minneapolis, MN, NY, USA) according to the manufacturer’s instructions. Readings were taken at 10 min following the addition of the stop solution at a wavelength of 450 nm with correction at 540 nm (*n* = 5–6).

### 2.3. Bioactivity of bFGF-Laden Scaffolds

To demonstrate the bioactivity of bFGF-releasing scaffolds, the mitogenic effect of bFGF was quantified by the proliferation of mouse myoblasts (C2C12, ATCC, Manassas, VA, USA) and human endothelial cells (HMEC-1 CDC, Atlanta, GA, USA). Each cell type was cultured on bFGF-laden nanofibrillar scaffolds or control nanofibrillar scaffolds in growth media (Dulbecco’s Modified Eagle’s Medium, supplemented with 10% fetal bovine serum and 1% penicillin/streptomycin). After 4 days, the cells were fixed in 4% paraformaldehyde for immunofluorescence staining of the S-phase cell cycle marker, Ki67. Immunofluorescence staining was carried out based on previously published methods [20,30,31]. In brief, the samples were permeabilized in 0.5% Triton-X-100 and then blocked in 1% BSA. Ki67 primary antibody (EMD Millipore, Burlington, MA, USA) was incubated onto samples overnight, followed by the addition of Alexa Fluor-488 and -594 secondary antibodies (Fisher Scientific, Hampton, NH, USA). Total cell nuclei were visualized by Hoechst 33342 (Fisher Scientific). Images were taken by confocal microscopy (Zeiss LSM 710, Oberkochen, Germany). To quantify the percentage of Ki67-expressing cells, the images were first converted to grayscale and then watershed segmentation was applied to separate touching cells. The Ki67 ^+^ cells and total cells were counted using the particle counting tool (Image J, NIH, Bethesda, MD, USA). The Ki67 ^+^ cells were expressed as a percentage of the total cells (*n* = 3).

### 2.4. bFGF-Laden Scaffolds and Exercise in a Mouse Full-Thickness VML Model

C57BL/6 mice (male, 8 weeks old, Jackson Laboratories, Bar Harbor, ME, USA) underwent a bilateral induction of VML. The animals were anesthetized with isoflurane (2–3%) at an oxygen flow rate of 1 L/min. A longitudinal incision was made in the skin directly above the tibialis anterior (TA) to expose the muscle. The TA was dissected from the extensor digitorum longus (EDL). A spatula was placed in between the EDL and TA muscles, and then a 2-mm diameter biopsy punch was applied to produce two adjacent holes in the central region of the TA, creating a 4 mm × 4 mm × 2 mm full-thickness defect that was approximately 20% of the weight of the TA. Immediately after tissue ablation, scaffold bundles that contained either bFGF or vehicle control (BSA) were placed into the defect, and the sides of the ablation were approximated using an 8–0 suture (S&T). The skin was then closed with an 8–0 nylon suture. Animals were placed in a cage on top of a warming pad to recover. The animals received sustained-release buprenorphine (0.6–1.0 mg/kg, Zoopharm, Laramie, WY, USA) for analgesia along with antibiotics (5 mg/kg, Enroflox, Norbrook Inc., Overland Park, KS, USA).

After induction of VML, the mice were allowed to rest for 7 days before further randomizing to receive non-exercise or exercise regimens, leading to four treatment groups with the following denotations: (1) scaffold (control), (2) bFGF-laden scaffold (bFGF scaffold), (3) scaffold with voluntary caged wheel exercise; or (4) bFGF-laden scaffold with caged wheel exercise. The mice in exercise groups were placed into running wheel cages (Scurry Mouse running Wheel 80820S, Lafayette Instrument Co., Lafayette, IN, USA) from days 7 through 21 post-surgery for voluntary exercise. To track running habits, each caged wheel was attached to an electronic counter that recorded the time and distance traveled every 15 s (Scurry 17.9 software, Lafayette Instrument Co.), as was conducted previously [13,28]. The non-exercise groups remained in regular group housing. At 21 days after induction of VML, the mice underwent muscle force measurements before euthanasia. The TA muscles were explanted for cryosectioning and histological analysis. All animal studies were approved by the Institutional Animal Care and Use Committee at the Veterans Affairs Palo Alto Health Care System.

### 2.5. Optimization of the Delayed Exercise Regimen

To identify an optimal time after induction of VML to administer rehabilitative exercise, a separate animal study was performed in which C57BL/6 mice were allowed to run on caged wheels starting from the day of VML induction (*n* = 6). The running distances of the mice were recorded for 2 weeks to evaluate the post-injury timepoint in which running habits reach steady-state levels.

### 2.6. Muscle Physiology

On day 21, after induction of VML, the mice underwent TA force measurement testing. An incision was made longitudinally in the skin of the lower leg to expose the TA. The distal tendon of the TA was severed, and the muscle was partially dissected so that the surrounding muscles did not contribute to the measurements. The dissected end of the tendon was attached to a 5–0 suture and secured to the transducer (Whole Animal System—Rat and Mouse, Aurora Scientific, ON, Canada). A needle was placed through the leg above the patella to secure the knee. Two electrodes were placed touching but not penetrating the TA to stimulate the muscle directly for isometric contraction. The Instantstim function from Dynamic Muscle Control (DMC) LabBook software (Aurora Scientific, ON, Canada) was used to stimulate twitch contractions in the TA muscle using a pulse of 0.2 ms and a train frequency of 0.5 Hz. While the muscle was being stimulated at 2 s intervals, the current was slowly increased until the TA achieved maximal stimulation. After the current was optimized, the length of the muscle was slowly increased until the optimal resting length was obtained. A single twitch force was then performed with a pulse width of 0.2 ms per leg for analysis (*n* = 7–8).

### 2.7. Immunofluorescence Staining and Quantification of Tissue Cross Sections

The TA muscles were explanted for routine cryosectioning and hematoxylin and eosin (H&E) staining. Immunofluorescence staining of tissue samples was carried out by fixation in 4% paraformaldehyde, followed by permeabilization in 0.5% Triton-X-100 [32,33]. For visualizing myofiber borders and neuromuscular junction formation, sections were blocked in 1% normal goat serum and then incubated in primary antibodies for laminin (Abcam, 1:100, Cambridge, UK) and α-bungarotoxin (Invitrogen, 1:100, Waltham, MA, USA), respectively. Alexa Fluor 488 or 594 secondary antibodies were diluted at a 1:100 ratio and subsequently incubated with tissue samples. Total nuclei were visualized using Hoechst 33342 dye. Tiled images using 20× objectives were taken using an epifluorescence microscope (Keyence, BZ-X700, Osaka, Japan) under 20× objectives.

To quantify muscle regeneration at the periphery of the scaffold implants, the total number of laminin + myofibers with centrally located nuclei within 500 µm radial distance from the implanted scaffold’s periphery was quantified. The data were expressed as regenerating muscle density (#/mm^2^, *n* = 5). For the quantification of neuromuscular junction formation, the total number of α-bungarotoxin-expressing junctions were quantified within a 500-µm radial distance from the scaffold and expressed as neuromuscular junction density (#/mm^2^, *n* = 5).

### 2.8. Statistical Analysis

Statistical analysis was performed using an unpaired *t*-test for comparisons of two groups. For comparisons of three or more groups, a one-way analysis of variance (ANOVA) with post hoc Tukey’s adjustment (GraphPad Prism, San Diego, CA, USA) was performed. Data are shown as a mean ± standard deviation (SD). Significance was accepted at *p* < 0.05 (*). All graphs were created in Microsoft Excel. Sample size reflects per operated leg unless otherwise noted.

## 3. Results

### 3.1. Characterization of bFGF-Laden Nanofibrillar Scaffolds

**Figure 1:**
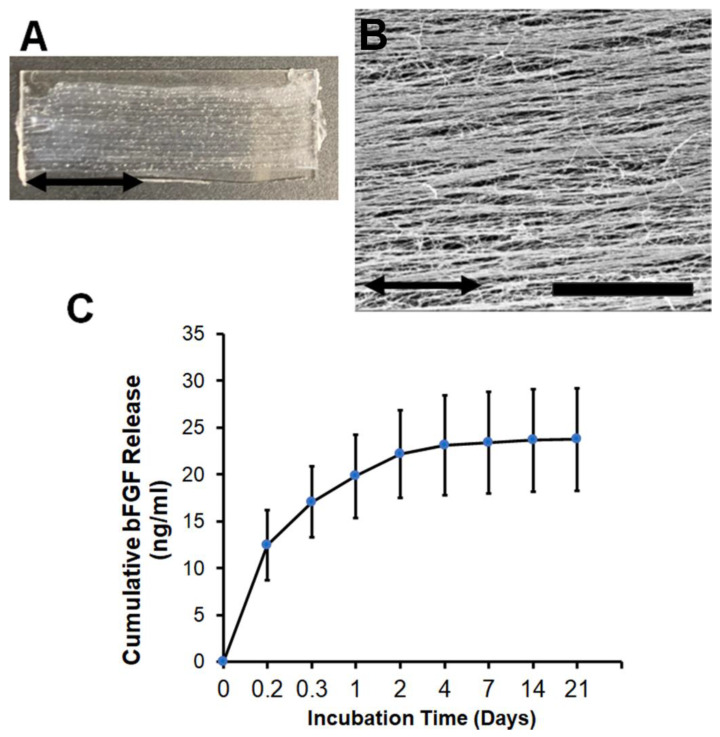
Aligned nanofibrillar scaffold fabrication and characterization. (**A**). Gross image of aligned nanofibrillar collagen scaffold bundle extruded and adhered onto a glass substrate. (**B**). Scanning electron microscopy image of aligned collagen scaffolds show organized nanofibrils in parallel. (**C**). Cumulative bFGF release from aligned nanofibrillar scaffolds over 21 days. Data are shown as mean ± SD (*n* = 5–6). Scale bar: 10 µm.

Aligned nanofibrillar scaffolds were fabricated by a facile shear-based extrusion technique, in which monomeric collagen was extruded from a syringe needle into a pH neutral saline, leading to rapid fibrillogenesis. The resultant collagen scaffolds were long and thin, with a scaffold width of approximately 300 µm. To create a scaffold bundle, 16 strips were immobilized adjacent to one another onto a glass substrate (Figure 1A). Owing to the application of shear during the extrusion process, fibrillogenesis of the monomeric collagen followed the direction of shear, leading to the formation of highly oriented collagen strips comprising nanoscale collagen fibrils (Figure 1B) as shown by scanning electron microscopy. The spatially organized collagen nanofibril structure is consistent with the findings from our previous publications [13,16,20,28]. For targeted local delivery of bFGF from the scaffolds, we chose the strategy of directly absorbing the bFGF into the scaffolds with overnight incubation, as this was previously shown to be a therapeutic strategy for the incorporation of other growth factors [28]. Furthermore, absorption approaches may have a more straightforward regulatory pathway for clinical translation compared to complex conjugation strategies. Adsorption of bFGF from the scaffolds led to an initial burst release of bFGF course during the first day, followed by a slower release of bFGF for the remaining 20 days (Figure 1C).

**Figure 2:**
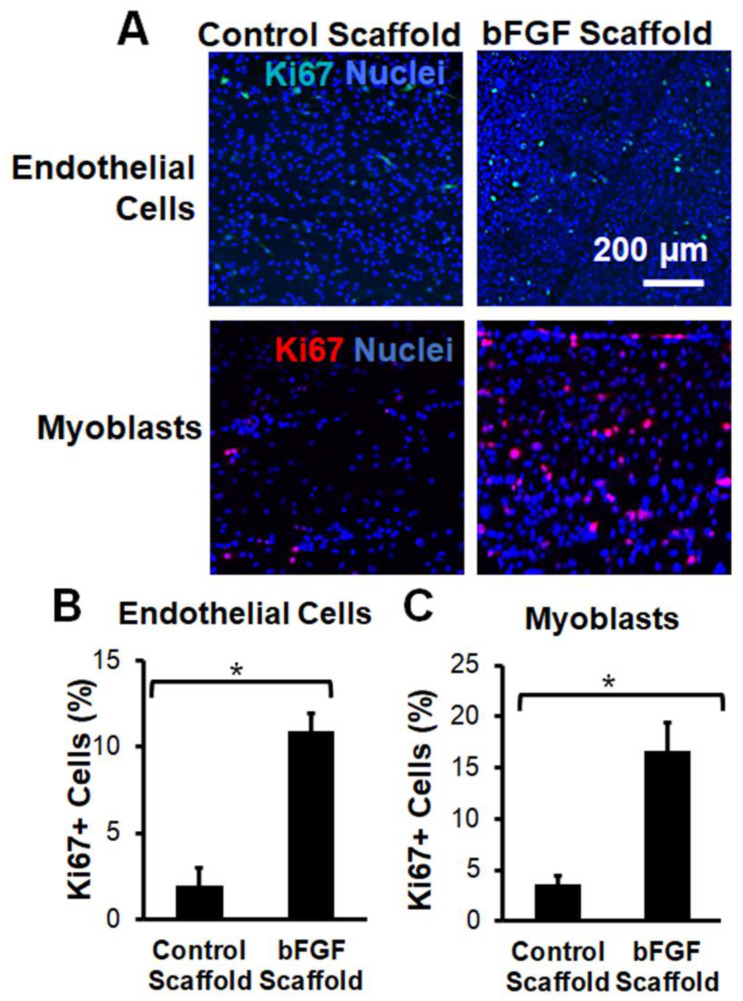
Bioactivity of bFGF-laden nanofibrillar scaffolds based on cellular mitogenic response. (**A**). Mouse myoblast (C2C12) and human endothelial (HMEC-1) cell lines cultured on the bFGF-laden scaffold or control scaffold for 4 days were immunofluorescently stained for Ki67. (**B**,**C**). Quantification of % Ki67^+^ endothelial cells (**B**) or myoblasts (**C**) show significant induction on the bFGF-laden scaffolds. Data are shown as mean ± SD (*n* = 3). * indicates *p* < 0.05.

To confirm the bioactivity of bFGF upon release from the scaffolds, we performed in vitro experiments to characterize the mitogenic response of skeletal muscle myoblasts or endothelial cells to bFGF stimulation. Skeletal myoblasts or endothelial cells were seeded onto the bFGF-laden scaffolds for 4 days before fixing the samples for immunofluorescence staining of the S-phase cell cycle marker, Ki67 (Figure 2A). Quantification of the percentage of total proliferating cells demonstrated a significant induction of cell proliferation upon culture on the bFGF-laden scaffolds. In particular, human endothelial cells showed a five-fold increase in cell proliferation in response to bFGF-releasing scaffolds, compared to scaffolds without bFGF (Figure 2B, *p* < 0.05). Additionally, skeletal myoblasts showed nearly a five-fold increase in proliferation on bFGF-laden scaffolds, compared to scaffolds without bFGF (Figure 2C, *p* < 0.05). These studies demonstrated that the bFGF released from the scaffolds were bioactive in inducing cellular proliferation.

### 3.2. Implantation of Aligned Nanofibrillar Scaffolds into the Site of VML

**Figure 3:**
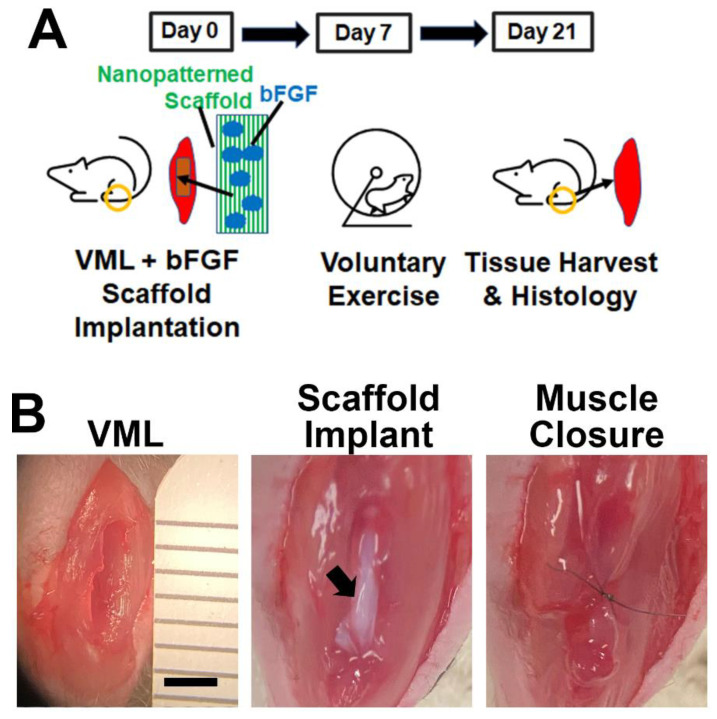
Overview of experimental design and surgical procedure. (**A**). Timeline of the study showing induction of VML and scaffold implantation on day 0. On day 7, specified treatment groups received caged wheel voluntary exercise for 2 weeks. On day 21, the tissues were harvested for histology. (**B**). Surgical images show full thickness VML, scaffold implantation, and muscle closure over the scaffold. Arrow denotes scaffold. Scale bar: 2 mm.

Upon validating the bioactivity of the bFGF-laden scaffolds in vitro, we next tested the therapeutic efficacy of bFGF-laden scaffolds in a murine model of VML. The bFGF-laden scaffolds were manually detached from the glass substrates to form a delaminated bundle of 16 scaffolds. The mice underwent bilateral full-thickness ablation of the TA muscle, followed by acute implantation of the bFGF-laden scaffold or the control scaffold into the site of muscle ablation (Figure 3A,B). The muscle was then sutured closed to immobilize the scaffold implant. To compare the efficacy of bFGF release to that of voluntary caged wheel exercise, additional animals received control scaffold implants and were allowed voluntary running in caged wheels at 7 days after scaffold implantation. Finally, to assess for interaction effects between bFGF and exercise, one treatment group received both bFGF-laden scaffold implants as well as rehabilitative exercise. All animals underwent terminal muscle physiology and tissue harvest 21 days after VML induction (Figure 3A).

The selection of a 7-day delay for voluntary caged wheel running was based on a study in which animals were allowed to undergo caged wheel running immediately after VML induction. Based on their daily running habits, we observed an unusually high degree of running distance on day 1, presumably due to the effect of post-surgery analgesics that masked the pain associated with running (Figure S1). Starting from day 2 after induction of VML, we observed an expected decline in running distance relative to day 1 and then a progressive increase in the total running distance per subsequent day. However, after 7 days from the time of VML induction, the progressive increase in the running distance became less pronounced, which suggested that the animals were approaching a plateau in their running distance. For this reason, we selected 7 days as the time to reach equilibrium running distance.

### 3.3. bFGF-Laden Scaffolds Induced Muscle Function Improvement

**Figure 4:**
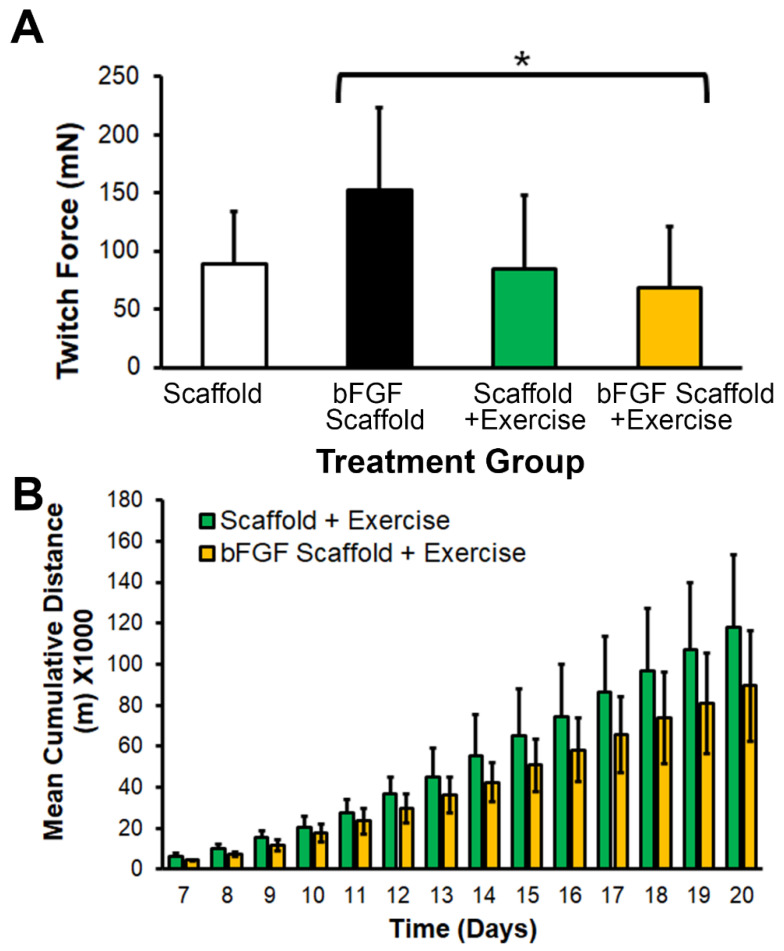
Functional effects of bFGF or voluntary exercise on muscle physiology or running distance. (**A**). Twitch force measurements of individual legs at 21 days after induction of VML (*n* = 7–8). * denotes a statistically significant relationship between bFGF scaffold vs. bFGF scaffold + exercise treatment groups (*p* < 0.05). (**B**). Mean cumulative running distance starting from 7 days after induction of VML (*n* = 4 animals). Data are shown as mean ± SD.

After 21 days of scaffold implantation, terminal muscle physiology was performed to assess the muscle function in all treatment groups. Intriguingly, the twitch force in mice treated with the bFGF-laden scaffold (152 ± 72 mN) was significantly higher than in animals treated with the combination of the bFGF-laden scaffold with exercise (69 ± 52 mN), whereas exercise alone appeared to not have led to significant improvements in twitch force (Figure 4A, *p* < 0.05). To assess for differences in running habits of animals with implants of the control scaffold or bFGF-laden scaffolds, the daily running distance was recorded for 21 days after induction of VML. The voluntary running habits showed a cumulative increase in running distance over the course of 21 days for animals treated with control scaffolds or bFGF-laden scaffolds, but no significant differences between these two exercise groups (Figure 4B). These data suggested that exercise did not lead to differences in running habits, but the treatment of bFGF-laden scaffolds improved muscle function.

**Figure 5:**
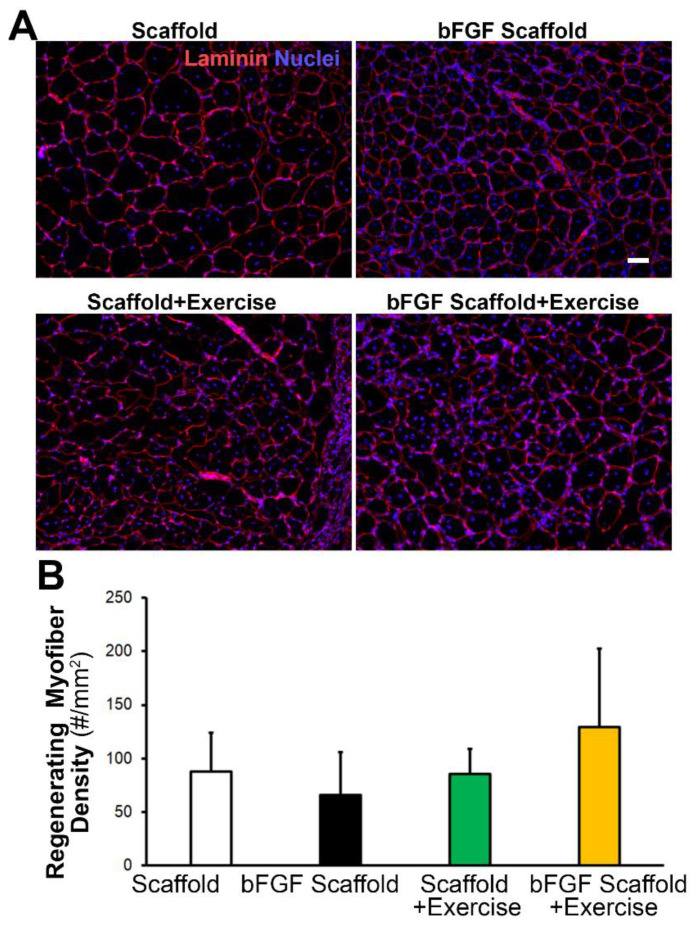
Effect of bFGF or exercise on myogenesis, based on tissue histology. (**A**). Fluorescence microscopy images adjacent to the site of scaffold implantation depict myofibers based on laminin (red) peripheral staining. (**B**). The regenerating muscle fiber density adjacent to the scaffold implant is quantified as the number of laminin^+^ myofibers with centrally located nuclei within 500 µm from the periphery of the scaffold. The density is expressed as the # of myofibers/mm^2^. Shown are mean ± SD (*n* = 4–5). Scale bar: 50 μm.

To further examine the effect of bFGF or exercise on muscle regeneration by tissue histology, the TA muscles were explanted after 21 days for routine cryosectioning, followed by hematoxylin and eosin (H&E) staining. From the H&E staining, the partially degraded collagen scaffold could be observed within the ablated muscle region, based on its characteristic ribbon-like fibrillar appearance (Figure S2). Regardless of bFGF incorporation, the scaffolds attracted cellular infiltration at the periphery of the scaffold, and to a lesser extent, less cellular infiltration in the center of the scaffold bundle. Immunofluorescence staining was next carried out to quantify the effects of bFGF or exercise on muscle myogenesis. Using an antibody directed against laminin that outlines the borders of myofibers, we visualized the newly formed myofibers based on the appearance of centrally located nuclei with laminin-expressing borders (Figure 5A). To determine whether scaffold-mediated bFGF release led to localized effects on tissue myogenesis, we quantified the density of newly formed myofibers within 500 µm radial distances from the scaffold bundle’s periphery (Figure 5B). Histological analysis of regenerating myofiber density composed of laminin + borders and centrally located nuclei did not have a significant effect among the treatment groups. Together, these data suggest that aligned nanofibrillar scaffolds promote cellular infiltration, but only bFGF-releasing nanofibrillar scaffolds significantly improved muscle function in a full-thickness muscle ablation model.

### 3.4. Scaffold Implantation with Exercise Promotes Re-Innervation

**Figure 6:**
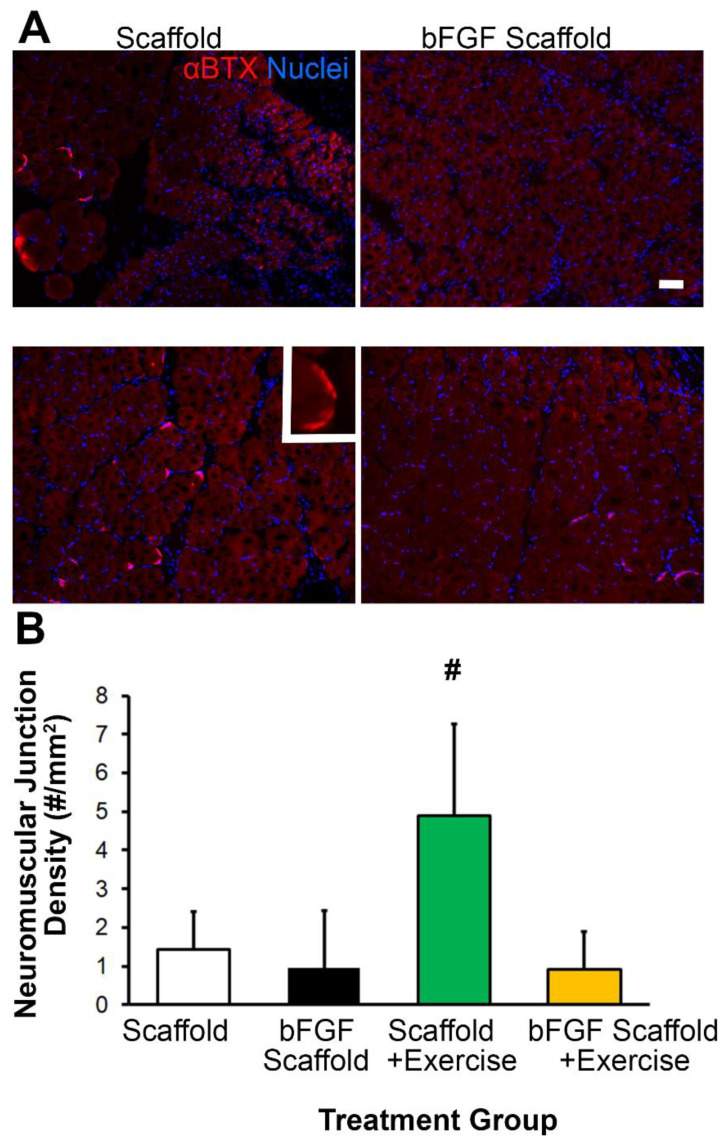
Effect of bFGF or exercise on re-innervation at the site of VML. (**A**). Representative microscopy images adjacent to the site of scaffold implantation depict nerve regeneration, based on α-bungarotoxin (αBTX) staining (red). Inset shows a magnified view of αBTX staining. (**B**). Quantification of neuromuscular junction density as the total number of neuromuscular junctions (αBTX) divided by the area formed by a 500-μm radial distance surrounding the scaffold. Shown are mean ± SD (*n* = 5). ^#^ denotes statistically significant comparison to all other groups (*p* < 0.05). Scale bar: 50 μm.

Since innervation is an important component of muscle regeneration, we quantified the density of neuromuscular junction formation by fluorescence staining (Figure 6A). Treatment of scaffold implantation with exercise led to the highest density of neuromuscular junctions (5 ± 2/mm^2^), compared to the scaffold treatment group (1 ± 1/mm^2^, *p* < 0.05). Interestingly, the combined treatment of bFGF-laden scaffold with exercise did not show any significant difference compared to the control group (Figure 6B). This data suggests that exercise, rather than bFGF, was a potent inducer of re-innervation.

## 4. Discussion

The salient findings of this research are that (1) bFGF and voluntary caged wheel exercise have differential effects on the treatment of VML; and (2) treatment of scaffolds releasing bFGF significantly improved muscle physiology after 3 weeks, whereas caged wheel exercise promoted neuromuscular junction formation. VML is a traumatic injury in which improved treatment strategies are in high demand. The delivery of pro-regenerative factors such as bFGF is a promising off-the-shelf therapy that can be applied in even austere settings. Since bFGF has been demonstrated to be safe in clinical trials for other indications such as wound healing and peripheral arterial disease [34,35], it is likely that bFGF will be safe for clinical treatment of VML.

To account for the large volume of muscle defect, an aligned nanofibrillar scaffold was implanted to the scaffold for the spatially controlled release of bFGF as well as for promoting cellular infiltration into or near the scaffold. Collagen was selected because of the ease of fabricating aligned nanofibrillar scaffold bundles using a highly facile extrusion process [19,20,29]. Nanofibrillar collagen scaffolds with parallel-aligned orientation can withstand higher loading when compared to conventional scaffolds that lack anisotropic collagen fibril organization [13]. In previous research, we further demonstrated the ability of aligned nanofibrillar collagen scaffolds to promote longitudinally aligned myofiber formation after induction of VML [16]. For these reasons, aligned nanofibrillar collagen scaffolds are well-suited for the treatment of VML.

In recognition that rehabilitative strategies can augment the benefits of tissue regeneration, we compared the effect of voluntary caged wheel running to that of localized bFGF release from nanofibrillar collagen scaffold. Similar to our previous findings using a partial thickness TA muscle ablation model, voluntary caged wheel running stimulation and the implantation of aligned nanofibrillar scaffolds promoted neuromuscular junction formation [13]. Our finding agrees with a larger body of work that physical exercise promotes re-innervation upon traumatic muscle injury [26,36].

Our results also show that the interaction between bFGF and voluntary caged wheel exercise is complex. For example, the implantation of bFGF-releasing scaffolds significantly increased twitch force generation (Figure 4A) compared to the combination of the bFGF-releasing scaffold with exercise. Conversely, the nanofibrillar scaffold implant with exercise promoted neuromuscular junction formation (Figure 6), but the combination of bFGF-releasing scaffold and exercise showed no improved re-innervation. These findings suggest that the timing, dosing, and duration of exercise and bFGF treatments may need to be further optimized for a therapeutic benefit. Therefore, it is possible that a longer duration of exercise might be necessary for observing therapeutic benefits in muscle regeneration. Additionally, published findings suggest that proper tuning of signaling pathways mediated by bFGF and exercise are necessary. For example, although bFGF and exercise can induce muscle satellite cell proliferation by the activation of signal transducer and activator of transcription 3 (STAT3) signaling [37,38], the tuning of STAT3 expression is critically important, as prolonged STAT3 expression has opposing effects of inducing muscle atrophy [39,40]. These points warrant further future investigation.

Although bFGF-releasing scaffolds could significantly improve muscle physiology, histological analysis of regenerating myofiber density did not reveal a statistically significant increase in regenerating myofiber density. Since the twitch force measurements quantitatively reflect the global function of the entire muscle, whereas histological methods semi-quantitatively reflect muscle regeneration in smaller regions of interest, it is plausible that the findings between both assays might contrast. However, the global nature of muscle force quantification possibly suggests a more reliable metric than tissue histological quantification.

## 5. Conclusions

In summary, we demonstrated that bFGF-laden aligned nanofibrillar collagen scaffolds for the treatment of VML in full-thickness muscle ablation model led to significantly improved muscle physiology, whereas collagen scaffold implants with voluntary caged wheel exercise promoted greater neuromuscular junction formation. Intriguingly, the combination of bFGF-laden scaffolds with rehabilitative exercise showed no therapeutic effect on the treatment of VML. Further studies to explore the optimal timing, dosing, and duration of exercise and bFGF treatments for therapeutic efficacy are warranted. This study highlights the differential therapeutic benefits of these two therapies separately, but not when prescribed in combination.

## Data Availability

The data presented in this study are available on request from the corresponding author.

## Supplementary Materials

The following are available online at https://www.mdpi.com/article/10.3390/bioengineering9010037/s1, Figure S1: Voluntary caged wheel running distance in mice starting on day 0 after induction of VML. Figure S2: Representative histological images of bFGF scaffold implants at 3 weeks after induction of VML.
