# Supplementary material for: Comparative Effects of Basic Fibroblast Growth Factor Delivery or Voluntary Exercise on Muscle Regeneration after Volumetric Muscle Loss
Source: Bioengineering (Basel). 2022 Jan 14;9(1):37. doi: 10.3390/bioengineering9010037 (PMC8773127; doi:10.3390/bioengineering9010037)
Supplement: Supplementary file 1 [file bioengineering-09-00037-s001.zip › bioengineering-1537921-supplementary.pdf]

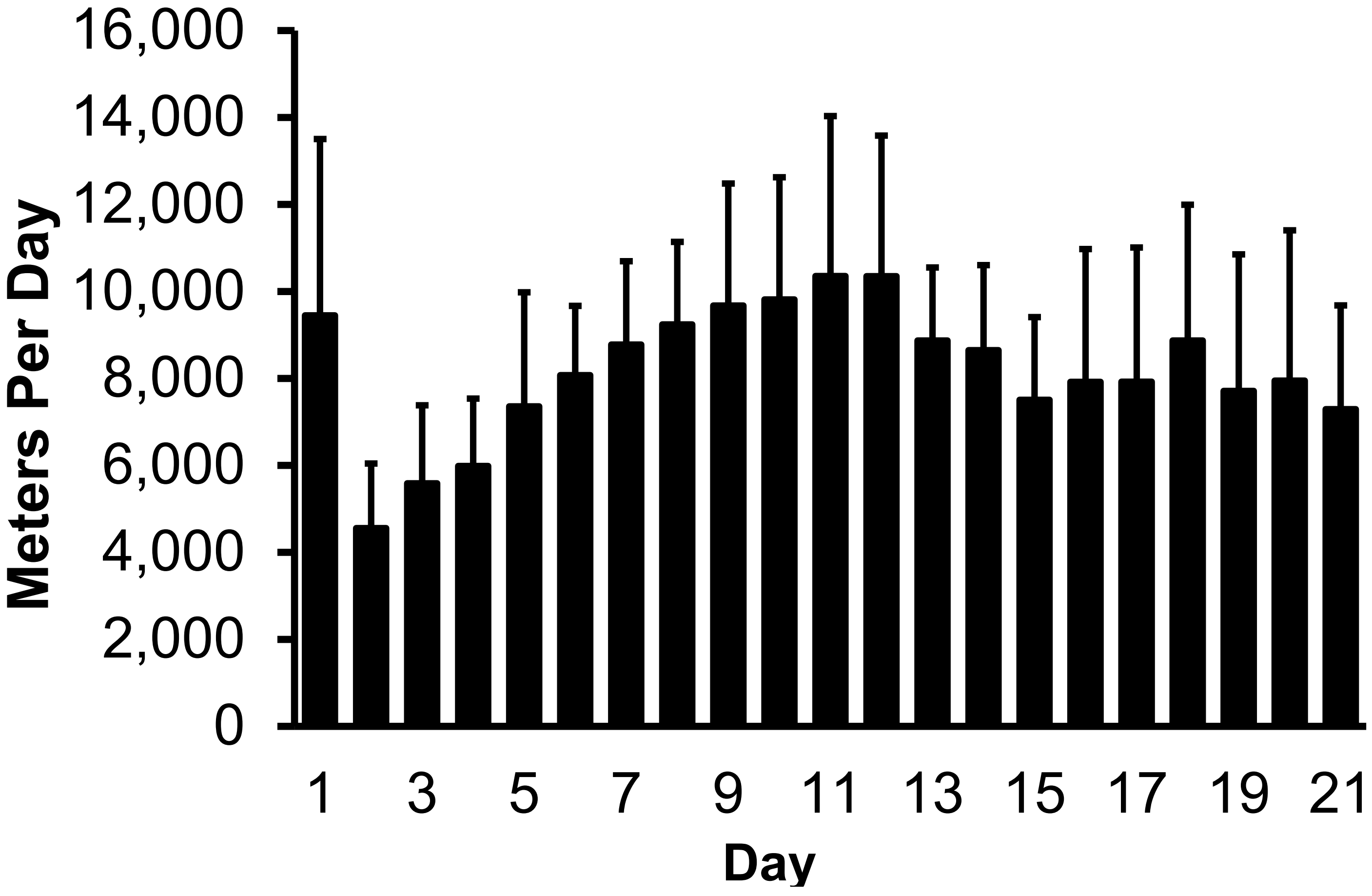

Figure S1. Voluntary caged wheel running distance in mice starting on day 0 after induction of VML. Data is shown as mean ± SD (n=6).

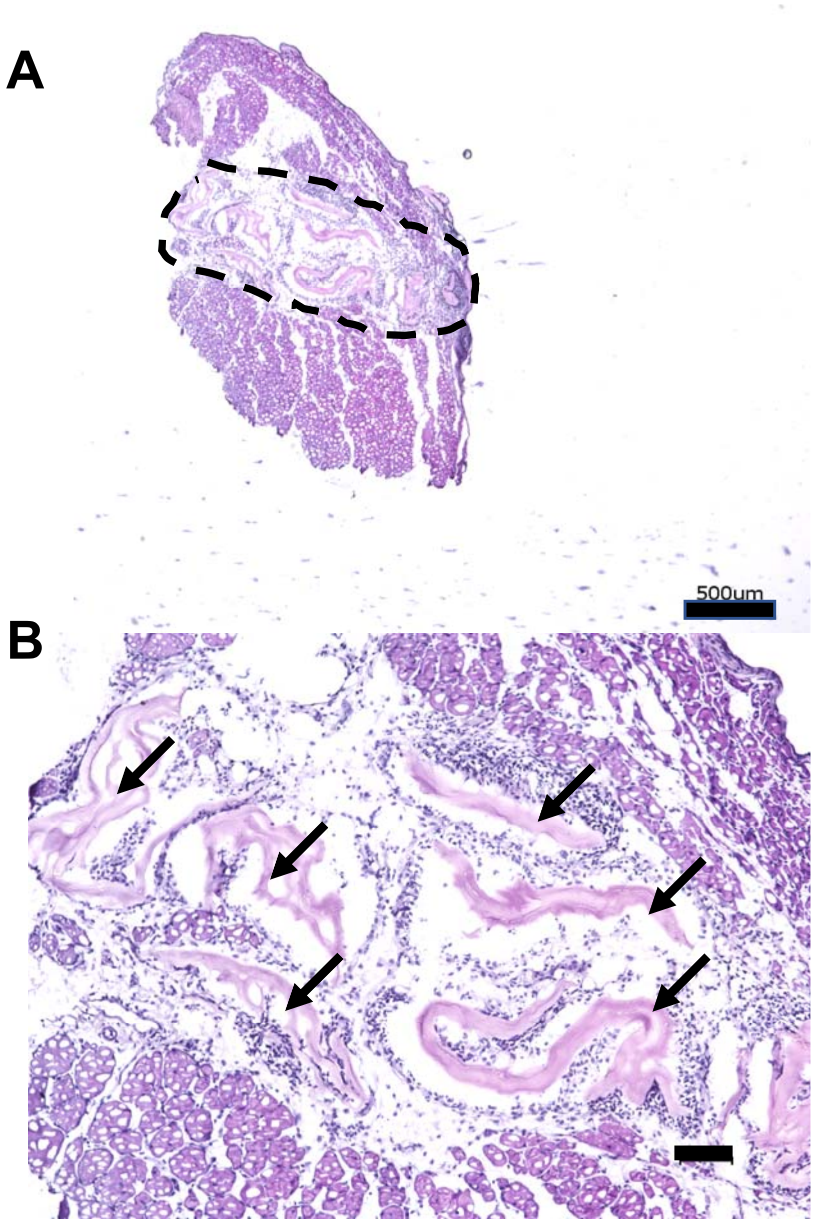

Figure S2. Representative histological images of bFGF scaffold implants at 3 weeks after induction of VML. **(A).** Low magnification image (2X objective) of scaffold implant, as shown by the dotted region in the hematoxylin and eosin stain. (**B**). Higher magnification image (10X objective) shows cellular infiltration and partial degradation of the collagen scaffolds, as indicated by the arrows. Scale bars: 500 μm **(A)** and 100 μm **(B)**.
